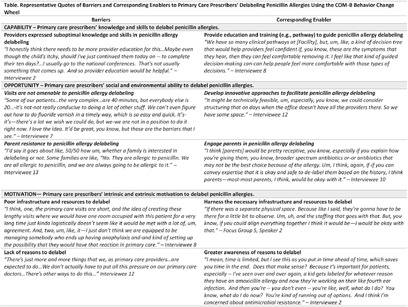# Barriers and Enablers to Penicillin Allergy Delabeling in Pediatric Primary Care: Findings from a Multisite Qualitative Study

**DOI:** 10.1017/ash.2025.254

**Published:** 2025-09-24

**Authors:** Eileen Carter, Elizabeth Monsees, Sharon Hwang, Tara Schmidt, Mary Lou Manning, Cliff O’Callahan, Rana El Feghaly, Monika Pogorzelska-Maziarz

**Affiliations:** 1 University of Connecticut School of Nursing; 2 Children’s Mercy Hospital; 3 Nemours Children’s Hospital; 4 Thomas Jefferson University; 5 Middlesex Health and University of Connecticut; 6 Children’s Mercy Kansas City; 7 Villanova University

## Abstract

**Background:** Up to 10% of children have penicillin allergy labels, although, when tested, >95% tolerate penicillin. These labels expose children to increased risks of harm through adulthood. Professional allergy societies recommend the proactive removal of low-risk penicillin allergy labels among children by history alone or following direct oral drug challenges. However, access to subspecialty allergy testing is limited and recent studies have demonstrated that direct oral amoxicillin challenges in low-risk populations can be safely performed in pediatric primary care settings. We aimed to identify prescribers’ attitudes towards penicillin allergy delabeling and barriers and enablers to penicillin allergy delabeling in pediatric primary offices. **Method:** We conducted a multisite qualitative study consisting of interviews and/or focus groups with 29 primary care prescribers at 10 primary care practices of two health systems in the northeast U.S. We analyzed data using conventional content analysis and grouped barriers and enablers to penicillin allergy delabeling according to the Capability, Opportunity, and Motivation domains of the COM-B Behavior Change Wheel. **Results:** Prescribers agreed that unnecessary penicillin allergy labels in children should be avoided and shared their experiences delabeling penicillin allergies from history alone and collaborating with parents to trial amoxicillin in children with low-risk penicillin allergies. Predominant barriers among prescribers to penicillin allergy delabeling included insufficient capability (suboptimal knowledge and skills in penicillin allergy delabeling), poor social and environmental opportunity (parent unwillingness to trial penicillin, lack of time, inadequate office space and resources), and poor motivation (tendency to accept reported penicillin allergies due to perception that consequences of penicillin allergy are rare and distant, inherent logistical difficulties to delabel, and lack of reasons to delabel). To facilitate penicillin allergy delabeling, participants recommended the implementation of a protocol and training in penicillin allergy delabeling, interventions to engage parents in delabeling, innovative approaches to address insufficient resources and infrastructures, and amplification of reasons for primary care prescribers to delabel. We provide representative quotes of the barriers and corresponding enablers to penicillin allergy delabeling in pediatric primary care in Table. **Conclusion:** There is precedent for penicillin allergy delabeling in pediatric primary care. Findings indicate that prescribers are inclined to delabel low-risk penicillin allergies if given the necessary education/training, parent support, resources, and infrastructure.

**Figure:**